# The effect of dietary education on ADHD, a randomized controlled clinical trial

**DOI:** 10.1186/s12991-015-0050-6

**Published:** 2015-03-01

**Authors:** Ahmad Ghanizadeh, Behzad Haddad

**Affiliations:** Research Center for Psychiatry and Behavioral Sciences, Department of Psychiatry, School of Medicine, Shiraz University of Medical Sciences, Shiraz, Iran; Department of Psychiatry, School of Medicine, Shiraz University of Medical Sciences, Shiraz, Iran; Department of Neuroscience, School of Advanced Medical Sciences and Technologies, Shiraz University of Medical Sciences, Shiraz, Iran

**Keywords:** ADHD, Attention, Diet, Clinical trial, Sugar, Cacao, Artificial food color

## Abstract

**Background:**

The purpose of this research was to study the effectiveness of the overall dietary intervention rather than a single nutrient on children with attention deficit hyperactivity disorder (ADHD).

**Methods:**

This is a randomized controlled trial conducted at a child psychiatry clinic in Iran. Participants were 106 children and adolescents with ADHD.

One group received methylphenidate plus dietary recommendations, while the other group only received methylphenidate. ADHD DSM-IV checklist was used to assess inattentiveness and hyperactivity/impulsivity scores at baseline and at the end of the trial.

**Results:**

The results revealed no significant difference between the two groups regarding mean age, gender ratio, body mass index, baseline inattentiveness score, and baseline hyperactivity score. Linear regression analysis considering the covariant variables showed that the inattentive score at the end of the trial was significantly associated with the mean change of favorite diet scores.

**Conclusion:**

This is the first clinical trial examining the effect of overall dietary characteristics rather than a single nutrient on the children formally diagnosed with ADHD. According to the results, un-favorite diet had no effects on inattentive or hyperactivity/impulsivity score. Encouraging the children with ADHD to increase their intake of recommended diet markedly improves their attention.

**Trial registration:**

The trial was registered at the Iranian Clinical Trials Registry (Irct ID: IRCT201311303930N29).

## Introduction

Attention deficit hyperactivity disorder (ADHD) is a common psychiatric disorder in children and adolescents. The neurobiology of ADHD is not clearly known. However, claims have been made that diet is associated with ADHD and infantile malnutrition causes long-lasting attention problems [1]. However, there is no scientific evidence showing that diet causes ADHD.

There are also some reports about the association of junk food [2], “Western” dietary pattern [3], and fast food [4] with ADHD symptoms.

Sweets and sugar are also believed to be associated with ADHD symptoms [4,5]. However, this assumption needs to be examined since some studies have not shown any association between sugar and behavioral problems [6,7]. On the contrary, another study reported that less consumption of sugar from fruit snacks increased the risk of ADHD [8]. Moreover, none of the studies has examined the effect of sugar on ADHD, but they have assessed its association with behavioral problems and cognition [6,7].

A 21-day, double-blind, placebo-controlled, repeated-measure study showed that ingestion of a synthetic food coloring was associated with irritability, restlessness, and sleep disturbance [9]. Toxicological, “anti-nutritional,” and hypersensitivity are the three supposed possible mechanisms for the effect of artificial food colors on the children’s behaviors [10]. However, evidence-based information is needed to support the potential efficacy of restricted diet on ADHD symptoms [11,12]. In a randomized controlled clinical trial, artificial food colorings and a preservative did not show any effects on the children’s behavior [13]. On the other hand, a systematic review showed that free fatty acid supplementation slightly reduced the ADHD symptoms and that exclusion of artificial food colors might reduce the symptoms in those with food sensitivities [14].

One other study reported that food supplements might be as effective as methylphenidate in improvement of some ADHD symptoms [15]. Nonetheless, more evidence regarding the efficacy of restricted diets is needed [14]. Although omitting some items is supposed to be helpful, their efficacy has not been assessed in controlled clinical trials [16]. In fact, it is a neglected research area [17]. Moreover, current literature suffers from publication bias and small non-generalizable samples [18]. Thus, further investigations are recommended to be conducted on the effect of diet on ADHD [18].

One study showed that intake of sweetened desserts, fried food, and salt are associated with more attention and behavioral problems while a balanced diet, regular meals, and a high intake of dairy products and vegetables are associated with less attention and behavioral problems [19]. However, the cross-sectional design of that study did not allow the authors to investigate a cause and effect relationship [19].

Moreover, as mentioned above, the current literature commonly associated some dietary factors with ADHD, while the overall dietary characteristics have been less taken into account [4]. Also, no studies have assessed the effect of overall dietary intervention on ADHD. This implies that assessment of the effect of overall diet rather than a single nutrient could be more informative [4]. Therefore, we designed a trial to examine the effect of these recommended variables on the children with ADHD. This is the first randomized controlled clinical trial investigating the adjuvant effect of “un-favored food diet” and “favored food diet” on treatment of the children and adolescents diagnosed with ADHD.

## Methods

The present trial was conducted on a group of children diagnosed with ADHD. The diagnosis was made through face-to-face interviews with the children and their parents and considering their teachers’ reports. The diagnosis was made according to DSM-IV diagnostic criteria supported by KSADS [20].

Assessments for both groups were performed at baseline and 1 month after the onset of the trial.

The patients were from both genders aged 5 to 14 years old with ADHD. Those with a serious medical condition or lack willingness to enter this trial were not entered. The patients/caregivers need to provide written informed consent.

ADHD severity was measured using ADHD checklist [21]. This checklist consists of 18 items, including 9 items for inattentiveness and 9 items for hyperactivity/impulsivity. The items are in fact the symptoms of ADHD according to DSM-IV. The instrument is scored through a Likert-type scale. The scores of inattentiveness and hyperactivity/impulsivity range from 0 to 27 with higher scores representing worse conditions.

### Anthropometric measurements

The children’s weight, height, and body mass index were measured.

### Dietary intake

Dietary intake was reported by the parents using a food frequency questionnaire for the last 1 month [4]. It was asked based on the nutrient similarity of foods. The food groups and their items were developed by a previous study on an Iranian sample [4]. According to that study, some examples of healthy foods are fruits, vegetables, whole grains, and dairy. On the other hand, some un-favored foods include sugar, soft drinks, commercially produced fruit juices, and sauces. Also, one of the items asked about the regular three meals a day intake. The parents in both groups reported the frequency of foods intake through a Likert-type scale ranging from never (0) to almost always (4).

We provided two lists of foods the first of which including the foods which were recommended to be eaten [4]. Some examples of these foods were diary, homemade fruit juices, vegetables, and low-fat meat. The other list included the foods which were recommended to be eaten as less as possible [4]. Some examples of this list were sugar, soft drinks, commercially produced fruit juices, and sauces. We called the first list as the “Favored foods” and the second one as “Un-favored foods.” In addition, the list requested the parents to provide their children with three regular meals.

### Groups

In this study, the patients were randomly allocated into two groups using a random number generator. Both groups received methylphenidate. However, one group was provided the list of favored and un-favored foods. Besides, one researcher explained the items of these lists to the parents. The other group, however, only received the medication.

The study was approved by the Ethics Committee of Shiraz University of Medical Sciences. In addition, written informed consents for taking part in the study were obtained from the patients or their parents. This trial was performed in accordance with the Declaration of Helsinki 1975.

### Statistical analysis

All the statistical analyses were performed using the SPSS statistical software. The categorical variables, such as gender ratio, were compared between the two groups using chi-squared test. In addition, *t*-test was used to compare the continuous variables, including age, body mass index, mean score of inattentiveness, and mean score of hyperactivity/impulsivity, between the two groups. Besides, the two groups were compared regarding the mean differences of favored food and un-favored food scores using *t*-test. The mean differences of inattentiveness and hyperactivity/impulsivity scores were also compared through *t*-test.

In the second round of the statistical analyses, two separate linear regression analyses were performed using Enter method. The degrees of changes in favorite and un-favorite foods were considered as continuous variables. Moreover, group, gender, body mass index, dose of methylphenidate intake at the beginning of the trial, dose of methylphenidate intake during the trial, mean difference of favored food score, mean difference of un-favored food score, mean score of inattentiveness at baseline, and mean score of hyperactivity/impulsivity at baseline were considered as the independent variables (covariates). On the other hand, mean scores of inattentiveness and hyperactivity/impulsivity at the end of the trial were considered as dependent variables. *P* value less than 0.05 was considered as statistically significant.

## Results

**Figure 1:**
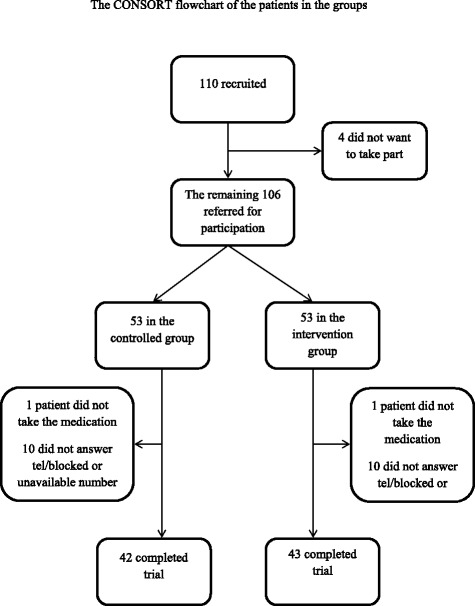
**The CONSORT flowchart of the patients in the groups.**

Among the 110 patients, 106 ones (53 in each group) agreed to participate in the study. However, only 43 patients in the treatment group and 42 patients in the control group received medication during the trial. One patient in each group refused to take the medications. In addition, ten patients in the control group and nine patients in the treatment group did not answer or blocked their phone calls. We are not aware if any patient dropped out due to the adverse effects of the medications (Figure 1).

The mean age of the patients in the treatment and control groups was 8.6(2.4) and 8.3(1.8) years, respectively (*t* = .76, *df* = 104, *P* = 0.4). Besides, 35 patients in the treatment group and 29 ones in the control group were male (*X*^2^ = 1.7, *df* = 1, *P* = 0.1). Moreover, 95.3% of the patients in the treatment group and 92.9% of those in the control group had taken methylphenidate during the month before entering the trial (*X*^2^ = .2, *df* = 1, *P* = 0.6). The mean dose of methylphenidate during the month before entering the trial was 9.7(7.2) and 9.3(2.9) mg/day in the treatment and the control group, respectively (*t* = .3, *df* = 98, *P* = 0.7). In addition, the mean dose of methylphenidate during the trial was 12.7(5.4) and 11.9(4.6) mg/day in the treatment and the control group, respectively (*t* = .7, *df* = 83, *P* = 0.4).

The inattentiveness score at baseline was not different between the treatment group and placebo group (15.7(6.0) versus 15.8(5.0); *t* = .08, *df* = 104, *P* = 0.9). The hyperactive/impulsivity score was not different between the two groups at baseline (17.7(6.0) versus 17.6(5.1); *t* = .03, *df* = 104, *P* = 0.9).

The findings of the present study revealed no significant difference between the two groups regarding the mean of body mass index (16.1(2.4) versus 15.4(2.6), *t* = 1.3, *df* = 102, *P* = 0.1).

The list of foods and dietary recommendations scores are reported in Table 1.

**Table 1:** **The mean (SD) score of the each of the items of diet in the groups**

**Items**	**Intervention group**		**Control group**	
	**Before**	**After**	**Before**	**After**
Favorite				
Takes at least two glasses of milk or other dairy products every day	3.2 (1.2)	4.2 (0.9)	3.1 (1.2)	3.2 (1.2)
Takes meat, fish, egg, or grains in each daily meal	3.5 (1.3)	4.3 (0.8)	3.7 (1.0)	3.7 (1.0)
Takes vegetables at each daily meal	2.6 (1.1)	3.3 (1.0)	2.5 (1.0)	2.6 (1.0)
Has fruit or homemade juices at least once a day	3.8 (1.2)	4.1 (1.1)	3.3 (1.3)	3.4 (1.4)
Has three regular meals a day	2.7 (1.4)	1.8 (0.9)	3 (1.1)	2.9 (1.2)
Does not eat what s/he wants	3.4 (1.5)	4.0 (1.3)	3.5 (1.5)	3.3 (1.5)
Takes cacao	3.2 (1.4)	2.1 (0.9)	3.0 (0.4)	2.9 (1.4)
Drinks coffee or tea	2.7 (1.3)	2.0 (1.0)	2.7 (1.4)	2.6 (1.3)
Un-favorite				
Takes fried foods more than twice a week	3.8 (1.1)	2.8 (1.2)	3.4 (1.0)	3.3 (1.1)
Eats fatty meat more than twice a week	2.5 (1.2)	1.9 (9.0)	2.5 (1.1)	2.3 (1.2)
Adds excessive salt or sauce to the foods	2.7 (1.4)	1.8 (0.9)	3.0 (1.1)	2.9 (1.2)
Eats ice cream, cake, or coke more than twice a week	4.1 (1.0)	2.9 (1.2)	4.2 (0.9)	4.0 (1.1)
Takes sugar and sweets	3.8 (1.2)	2.5 (1.0)	3.6 (1.1)	3.4 (1.10
Takes pickles	2.4 (1.3)	1.6 (1.0)	2.6 (1.3)	2.4 (1.2)
Takes chips, puffs, and factory made fruit juices	3.6 (1.3)	2.3 (1.1)	3.8 (1.1)	3.6 (1.1)

Responses to each item could range from never (0) to almost always (4).

The score of favorite diet in the treatment group increased from 25.7(4.6) to 28.3(4.4). However, the scored of favorite diet in the control group changed from 24.8(5.5) to 25.0(5.1) during this trial.

The mean of favorite diet score change was significantly different between the two groups (2.6(3.9) versus − .02(1.9) for the treatment and control group, respectively (*t* = 3.7, *df* = 78, *P* < 0.001) (Table 2). The score of un-favorite diet changed from 22.7(4.7) to 16.2(5.0) in the treatment group. It changed from 23.2(4.5) to 21.9(5.9) control groups, respectively. The mean of un-favorite diet score change was different between the two groups (*t* = 5.2, *df* = 78, *P* < 0.001).

**Table 2:** **Basic characteristics and mean differences of favored and un-favored foods scores in the two groups**

**Variable**	**Group**		**Significance**
	**Treatment**	**Control**	
	**Mean (standard deviation)**	**Mean (standard deviation)**	
Mean (SD) years of age	8.6 (2.4)	8.3 (1.8)	*t* = .76, *df* = 104, *P* = 0.4
Dosage of methylphenidate during the trial (mg/day)	12.7 (5.4)	11.9 (4.6)	*t* = .7, *df* = 83, *P* = 0.4
Dosage of methylphenidate before the trial (mg/day)	9.7 (7.2)	9.3 (2.9)	*t* = .3, *df* = 98, *P* = 0.7
Body mass index	16.1 (2.4)	15.4 (2.6)	*t* = 1.3, *df* = 102, *P* = 0.1
Inattentiveness score at baseline	15.7 (6.0)	15.8 (5.0)	*t* = .08, *df* = 104, *P* = 0.9
Inattentiveness score at the end	12.5 (5.3)	11.6 (5.8)	*t* = .7, *df* = 78, *P* = 0.4
Hyperactivity/impulsivity score at baseline	17.7 (6.0)	17.6 (5.1)	*t* = .03, *df* = 104, *P* = 0.9
Hyperactivity/impulsivity at end	13.9 (6.2)	13.1 (5.9)	*t* = .5, *df* = 78, *P* = 0.5
The mean difference of favorite diet during the trial	2.6 (3.9)	−.02 (1.9)	*t* = 3.7, *df* = 78, *P* < 0.0001
The mean difference of un-favorite diet during the trial	−6.5 (5.2)	−1.2 (3.7)	*t* = 5.2, *df* = 78, *P* < 0.0001
The mean difference of inattentiveness score during the trial	−3.3 (4.8)	−4.0 (5.1)	*t* = .7, *df* = 78, *P* = 0.4
The mean difference of hyperactivity/impulsivity score during the trial	−3.7 (4.7)	−5.0 (4.8)	*t* = 1.1, *df* = 78, *P* = 0.2

Also, no significant difference was found between the two groups concerning the mean change of hyperactivity/impulsivity and inattentiveness scores (Table 1). However, a significant negative correlation was observed between the inattentiveness scores at the end of the trial and the mean change of favorite diet scores (*r* = −0.24, *P* < 0.03). There was no significant correlation between inattentiveness score at baseline with baseline favorite or un-favorite diet scores (*P* < 0.5, *P* < 0.1; respectively). The score of hyperactivity/impulsivity at baseline was not associated with baseline favorite or un-favorite diet scores (*P* < 0.7, *P* < 0.9; respectively).

Linear regression analysis considering the covariant variables showed that the inattentive scores at the end of the trial were significantly associated with the mean change of favorite diet scores (Table 3). Nevertheless, no significant relationship was found between age, gender, dose of methylphenidate during the trial, body mass index, and mean change of un-favorite diet scores and the inattentiveness score at the end of the trial (Table 3).

**Table 3:** **Linear regression analysis for the association between the inattentiveness score at the end of the trial and the independent variables**

**Variable**	**Beta**	**Significance**	**95% confidence interval**	
			**Lower bound**	**Upper bound**
Age	−.004	.9	−.52	.50
Gender	−.09	.2	−3.46	1.01
Dosage of methylphenidate during the last month before entering this trial	.04	.6	−.14	.23
Body mass index	−.13	.1	−.71	.12
Favorite diet change score	−.31	.001	−.81	−.23
Un-favorite diet change score	−.15	.08	−.34	.02
Inattentiveness score at baseline	.66	.001	.49	.85

The second linear regression analysis considering the covariant variables indicated no significant association between the hyperactivity/impulsivity scores at the end of the trial and age, gender, dose of methylphenidate during the trial, body mass index, mean change of favorite diet scores, and mean change of un-favorite diet scores.

## Discussion

The current trial compared the effect of methylphenidate plus dietary intervention versus methylphenidate alone on the children and adolescents with ADHD. Performing the analyses considering the groups as categorical variables did not show any difference between the outcomes of the two groups. The degree of cooperation between the parents and children for applying the dietary recommendations is a continuous variable. Moreover, the patients might have performed the intervention differently. Some of them might have dramatically increased the favorite food intake, while some others might have not. Therefore, we performed the statistical analyses considering the dietary intervention as a continuous variable using the food questionnaire. Therefore, we included the degree of compliance to our recommendations as a covariate factor. In this analysis, the mean changes of favorite and un-favorite diet scores were calculated. This change of scores was considered as an independent variable in linear regression analysis. The most striking finding of this study was the negative correlation between the increase of favorite diet scores and the scores of inattentiveness severity at the end of the trial. However, the results of regression analysis showed that none of the other variables, i.e., age, gender, medication dosage, body mass index, and mean change of un-favorite foods score, was significantly associated with the inattentiveness scores. This implies that the children who markedly increased their intake of favorite foods experienced lesser inattentiveness in comparison to those with none or less increase of favorite diet intake. To the best of our knowledge, this is the first study which examined the effect of both favorite and un-favorite diets on the children with ADHD. We could not find any similar trials to compare our results.

The present study showed that un-favorite diet had no effects on inattentive or hyperactivity/impulsivity scores. Previous reports about the association between junk food [2] and “Western” dietary pattern [3] and ADHD were descriptive studies rather than interventional clinical trials. In addition, the findings regarding the relationship between sugar and ADHD symptoms are very controversial and many studies have not shown this relation [6,7]. Meanwhile, one study showed that less consumption of sugar from fruit snacks increased the risk of ADHD [8]. However, long-term trials are needed to examine the effect of un-favorite diet on inattention.

Overall, our results are in agreement with those of a descriptive study indicating that a balanced diet, regular meals, and a high intake of dairy products and vegetables were associated with less attention and behavioral problems [19].

Nevertheless, descriptive studies conducted on the effect of synthetic food colorings on attention were not conclusive [9]. For instance, one study was not a clinical trial and also evaluated the association between the food colorings and some behaviors rather than ADHD [9]. One other study also suggested that omitting some food items could be helpful; however, its efficacy has never been examined in controlled clinical trials [16].

This study had some limitations. First of all, the sample size was relatively small. The duration of the intervention was also relatively short. However, we were not sure if we could get the Ethic Committee’s approval due to the lack of any strong evidence about the usefulness and lack of negative consequences of the recommended diet on the children with ADHD. Moreover, although some of the covariant variables were taken into account by statistical adjustment, the patients were taking the medications concurrently. Therefore, they were stable of the medication. So, the dietary intervention was an adjuvant.

Another explanation is that children with better attention at baseline more successfully followed our recommendations. However, we considered this as a covariate factor in the regression analysis. Therefore, it does not seem that the effect of intervention of the attention score be just due to baseline attention score.

We also could not consider a control group including the patients with dietary intervention alone. In fact, there was no evidence that dietary intervention alone would be effective, and we probably were not able to convince the Ethics Committee to omit the medication in one group. Furthermore, sampling and the trial were performed in one season and included only a clinical sample from a university-affiliated child psychiatry clinic. Therefore, generalization of the results to other seasons or other clinics should be performed with caution. Further studies may also include objective biochemical measurements.

Nevertheless, this is the first randomized controlled clinical trial which examined the effect of overall dietary characteristics rather than a single nutrient on the children formally diagnosed with ADHD.

## Conclusion

Dietary recommendations by the investigators led to a significant improvement in diet compared to no intervention. However, the two experimental groups did not show different amounts of improvement in measures of attention. Nevertheless, when both groups were combined, there was a significant relationship between improvements in favorite dietary behavior and improvements in measures of attention. Un-favorite diet intake was not associated with improvements in either inattentiveness or hyperactivity/impulsivity.

## References

[1] Galler JR, Bryce CP, Zichlin ML, Fitzmaurice G, Eaglesfield GD, Waber DP (2012). Infant malnutrition is associated with persisting attention deficits in middle adulthood. J Nutr.

[2] Wiles NJ, Northstone K, Emmett P, Lewis G (2009). ‘Junk food’ diet and childhood behavioural problems: results from the ALSPAC cohort. Eur J Clin Nutr..

[3] Howard AL, Robinson M, Smith GJ, Ambrosini GL, Piek JP, Oddy WH (2011). ADHD is associated with a “Western” dietary pattern in adolescents. J Atten Disord.

[4] Azadbakht L, Esmaillzadeh A (2012). Dietary patterns and attention deficit hyperactivity disorder among Iranian children. Nutrition.

[5] Johnson RJ, Gold MS, Johnson DR, Ishimoto T, Lanaspa MA, Zahniser NR (2011). Attention-deficit/hyperactivity disorder: is it time to reappraise the role of sugar consumption?. Postgrad Med.

[6] Krummel DA, Seligson FH, Guthrie HA (1996). Hyperactivity: is candy causal?. Crit Rev Food Sci Nutr.

[7] Wolraich ML, Lindgren SD, Stumbo PJ, Stegink LD, Appelbaum MI, Kiritsy MC (1994). Effects of diets high in sucrose or aspartame on the behavior and cognitive performance of children. N Engl J Med.

[8] Kim Y, Chang H (2011). Correlation between attention deficit hyperactivity disorder and sugar consumption, quality of diet, and dietary behavior in school children. Nutr Res Pract.

[9] Rowe KS, Rowe KJ (1994). Synthetic food coloring and behavior: a dose response effect in a double-blind, placebo-controlled, repeated-measures study. J Pediatr.

[10] Stevenson J (2006). Dietary influences on cognitive development and behaviour in children. Proc Nutr Soc.

[11] Konikowska K, Regulska-Ilow B, Rozanska D (2012). The influence of components of diet on the symptoms of ADHD in children. Roczniki Panstwowego Zakladu Higieny.

[12] Arnold LE, Lofthouse N, Hurt E (2012). Artificial food colors and attention-deficit/hyperactivity symptoms: conclusions to dye for. Neurotherapeutics.

[13] Lok KY, Chan RS, Lee VW, Leung PW, Leung C, Leung J (2013). Food additives and behavior in 8- to 9-year-old children in Hong Kong: a randomized, double-blind, placebo-controlled trial. J Dev Behav Pediatr.

[14] Sonuga-Barke EJ, Brandeis D, Cortese S, Daley D, Ferrin M, Holtmann M (2013). Nonpharmacological interventions for ADHD: systematic review and meta-analyses of randomized controlled trials of dietary and psychological treatments. Am J Psychiatry.

[15] Harding KL, Judah RD, Gant C (2003). Outcome-based comparison of Ritalin versus food-supplement treated children with AD/HD. Altern Med Rev.

[16] Millichap JG, Yee MM (2012). The diet factor in attention-deficit/hyperactivity disorder. Pediatrics.

[17] Schnoll R, Burshteyn D, Cea-Aravena J (2003). Nutrition in the treatment of attention-deficit hyperactivity disorder: a neglected but important aspect. Appl Psychophysiol Biofeedback.

[18] Nigg JT, Lewis K, Edinger T, Falk M (2012). Meta-analysis of attention-deficit/hyperactivity disorder or attention-deficit/hyperactivity disorder symptoms, restriction diet, and synthetic food color additives. J Am Acad Child Adolesc Psychiatry.

[19] Park S, Cho SC, Hong YC, Oh SY, Kim JW, Shin MS (2012). Association between dietary behaviors and attention-deficit/hyperactivity disorder and learning disabilities in school-aged children. Psychiatry Res.

[20] Ghanizadeh A, Mohammadi MR, Yazdanshenas A (2006). Psychometric properties of the Farsi translation of the Kiddie Schedule for Affective Disorders and Schizophrenia-Present and Lifetime Version. BMC Psychiatry..

[21] Ghanizadeh A, Jafari P (2010). Cultural structures of the Persian parents’ ratings of ADHD. J Atten Disord.

